# Quantitative Detection of *Salmonella Typhimurium* in Ground Chicken Using a Surface Plasmon Resonance (SPR) Biosensor

**DOI:** 10.3390/bios15120814

**Published:** 2025-12-15

**Authors:** Sandhya Thapa, Fur-Chi Chen

**Affiliations:** Department of Food and Animal Sciences, Tennessee State University, Nashville, TN 37209, USA; sthapa1@my.tnstate.edu

**Keywords:** biosensor, surface plasmon resonance, *Salmonella*, monoclonal antibodies, food contamination, ground chicken

## Abstract

Regulatory agencies worldwide have implemented stringent measures to monitor and reduce *Salmonella* contamination in poultry products. Rapid quantitative detection methods enable producers to identify contamination early, implement corrective actions, and enhance food safety. This study aimed to develop and optimize a surface plasmon resonance (SPR) biosensor for the quantitative detection of *Salmonella Typhimurium* in ground chicken. The sensor surface was functionalized with a well-characterized monoclonal antibody specific to *Salmonella* flagellin, and an SPR workflow was established for quantitative analysis. Ground chicken samples were inoculated with four *S. Typhimurium* strains at contamination levels ranging from −0.5 to 3.5 Log CFU/g and enriched at 42 °C for 10 or 12 h prior to SPR analysis. Contamination levels were confirmed using the Most Probable Number (MPN) method. Linear regression analysis indicated that optimal quantification was achieved after 10 h of enrichment (R^2^ ≥ 0.86), whereas extended enrichment (12 h) did not improve performance. The limit of quantification (LOQ) was below 1 CFU/g. A strong positive correlation (R^2^ ≥ 0.85) was observed between SPR and MPN results, demonstrating consistency between the two methods. These findings highlight SPR as a rapid, reliable, and cost-effective alternative to conventional methods for *Salmonella* quantification. By delivering accurate results within a single day, SPR enhances testing efficiency and supports the production of safer poultry products, thereby reducing public health risks associated with *Salmonella* contamination.

## 1. Introduction

Non-typhoidal *Salmonella* is a leading bacterial pathogen responsible for foodborne illness in the United States, with over one million cases reported annually. It ranks as the third most common cause of foodborne illness and hospitalizations and is the foremost contributor to foodborne-related deaths [1]. According to the Centers for Disease Control and Prevention, *Salmonella* infections result in approximately 1.35 million illnesses, 26,500 hospitalizations, and 420 deaths each year [2]. The associated economic burden, including healthcare costs, productivity losses, and premature mortality, is estimated to range between $4 and $11 billion annually, disproportionately affecting vulnerable populations such as immunocompromised individuals, children, and the elderly [3,4,5]. The genus *Salmonella* comprises two species, *Salmonella enterica* and *Salmonella bongori*, both of which are capable of causing human illness [6]. These organisms are typically identified by their serotypes rather than species designation [7,8]. To date, over 2600 distinct serotypes have been characterized [9,10]. Among these, *Salmonella enterica* subsp. enterica serovar Typhimurium is the second most prevalent serotype implicated in foodborne outbreaks, following serovar Enteritidis [11]. *Salmonella* contamination spans a wide range of food products, including fresh produce, meat, and poultry. Given that poultry is the most consumed meat in the United States, its role as a reservoir for foodborne pathogens presents a significant public health concern [12]. Data from the Interagency Food Safety Analytics Collaboration (2022) indicates that poultry accounts for over 24% of foodborne *Salmonella* infections, with chicken alone responsible for more than 19% [13].

Various methodologies are available for the detection and quantification of *Salmonella*. Traditional culture-based techniques, such as the standard plate count method, remain the gold standard due to their simplicity, cost-effectiveness, and reliability [14,15]. However, these methods are labor-intensive and time-consuming, often requiring 2–3 days for preliminary identification and over a week for confirmation, limiting their utility for real-time pathogen detection [16,17]. Additionally, their sensitivity is compromised by the presence of viable but non-culturable cells, which may yield false-negative results [16,18].

Immunological assays, such as enzyme-linked immunosorbent assays (ELISA) and lateral flow immunoassays, offer faster detection and are valued for their simplicity and stability. Nonetheless, these methods often require pre-enrichment steps and complex sample preparation, and they may suffer from limited sensitivity and false-negative outcomes. Molecular techniques, particularly polymerase chain reaction (PCR)-based assays, provide superior speed, sensitivity, and specificity and are widely adopted [15]. Despite these advantages, PCR methods are generally qualitative, and their performance can be hindered by sample inhibitors, challenges in automation, inability to distinguish viable from non-viable cells, and the need for skilled personnel and precise primer design [5,19,20,21].

The Most Probable Number (MPN) method is another commonly used approach for bacterial quantification, relying on serial dilutions and multiple tube inoculations. While effective, its labor-intensive and time-consuming nature underscores the need for more rapid, reliable, and cost-efficient detection technologies [22,23].

Surface plasmon resonance (SPR) biosensors have emerged as a promising alternative for foodborne pathogen detection due to their real-time monitoring capabilities, high sensitivity and specificity, and label-free operation [24,25,26,27]. SPR operates on the principle of total internal reflection, detecting changes in refractive index to monitor biomolecular interactions such as antigen–antibody binding [28]. It is capable of directly detecting large targets like *Salmonella*, which have molecular weights exceeding 10 kDa [29]. The use of well-characterized monoclonal antibodies can significantly enhance the specificity and sensitivity of SPR assays by minimizing non-specific binding and cross-reactivity, issues commonly associated with polyclonal antibodies [30].

SPR biosensors have been applied in diverse contexts, including the detection of biomolecules, antibiotic residues, food adulterants, mycotoxins, genetically modified organisms, pesticides, herbicides, insecticides, and microorganisms [31]. Several studies have explored the use of SPR for detecting *Salmonella* in various food matrices [32,33,34,35,36,37,38,39,40,41,42,43]. However, to the best of our knowledge, no studies have reported the quantitative detection of *Salmonella Typhimurium* in chicken meat using SPR technology.

Therefore, the objective of this study was to develop and validate an SPR-based method for the quantitative detection of *Salmonella Typhimurium* in ground chicken and to compare its performance with the conventional MPN method.

## 2. Materials and Methods

### 2.1. Materials and Instruments

Four strains of *Salmonella enterica* serovar Typhimurium (ATCC 13311, ATCC 29629, ATCC 49416, and ATCC 59812) were obtained from the American Type Culture Collection (Manassas, VA, USA) and stored at −80 °C until use. Ground chicken samples were purchased from a local retail outlet to serve as the food matrix for contamination studies.

Culture media, including Tryptic Soy Agar (TSA), Tryptic Soy Broth (TSB), Xylose-Lysine-Tergitol 4 (XLT-4) agar, and Buffered Peptone Water (BPW), were procured from Thermo Fisher Scientific (Lenexa, KS, USA). Most Probable Number (MPN) media was supplied by Hygiena (Camarillo, CA, USA). Reagents for sensor surface chemistry—10× phosphate-buffered saline (PBS), Tween 20, N-(3-dimethylaminopropyl)-N′-ethylcarbodiimide hydrochloride (EDC), N-hydroxysuccinimide (NHS), ethanolamine hydrochloride, sodium acetate, and glycine—were obtained from Fisher Scientific (Fair Lawn, NJ, USA) and Sigma-Aldrich (St. Louis, MO, USA). Deionized water was prepared using a Millipore Simplicity purification system and degassed under vacuum prior to use; all solutions were prepared using this degassed water. The BAX^®^ Q7 system and *Salmonella* Quant kits were purchased from Hygiena for molecular quantification.

Surface plasmon resonance (SPR) assays were performed using the Reichert Dual Channel SR7500DC SPR System (Reichert Technologies, Buffalo, NY, USA) with Integrated SPRAutolink software (Version 1.1.14-T). Sensor data were analyzed using TraceDrawer software (Version 1.6.1; Ridgeview Instruments AB, Uppsala, Sweden). Sensor chips with a 500 kDa carboxymethyl dextran hydrogel surface (SR7000 gold sensor slides) were purchased from Reichert Inc (New York, NY, USA). Monoclonal antibodies (MAb 1E10) were synthesized in-house following protocols described by [41].

### 2.2. Preparation of SPR Sensor Surface

Sensor chips were mounted on the Reichert SR7500DC system according to manufacturer instructions. The sensor surface was preconditioned with PBST (1× PBS containing 0.05% TWEEN 20) at a flow rate of 20 μL/min until a stable baseline was achieved. Immobilization of monoclonal antibody MAb 1E10 was performed at 25 °C and 20 μL/min flow rate. A freshly prepared activation solution containing 40 mg EDC and 10 mg NHS in 1 mL deionized water was injected for 5 min to activate carboxyl groups on the sensor surface. MAb 1E10 (150 μg/mL in 10 mM sodium acetate, pH 5.2) was then injected into the left channel for 5 min. To block residual active sites, bovine serum albumin (BSA; 75 μg/mL in 10 mM sodium acetate, pH 5.2) was injected into both channels. Unbound reagents were removed and carboxyl groups quenched by injecting 1.0 M ethanolamine (pH 8.5) for 5 min. After immobilization, a continuous flow of PBST at 20 μL/min was maintained, and SPR assays were initiated once a stable baseline was established. All experiments were conducted at 25 °C.

### 2.3. Preparation of Positive Control for SPR

A positive control was prepared using *S. Typhimurium* ATCC 13311. A single colony from a TSA plate was inoculated into 10 g of ground chicken placed in a filter bag containing 225 mL BPW. The mixture was homogenized for 30 s using a Stomacher Circulator and incubated at 37 °C for 24 h. Aliquots (1 mL) of the enriched homogenate were stored at −80 °C in Eppendorf tubes for use as positive controls.

### 2.4. Preparation of Salmonella Cultures for Inoculation

Glycerol-preserved stocks of the four *Salmonella* enterica serovar Typhimurium strains were streaked onto Tryptic Soy Agar (TSA) plates and incubated at 37 °C for 22–24 h. Single colonies from each strain were transferred into 10 mL of Tryptic Soy Broth (TSB) and cultured overnight (approximately 18 h) at 37 °C. Equal volumes of the resulting cultures were combined to prepare a four-strain composite inoculum. The mixed culture was standardized to a target concentration of approximately 10^8^ CFU/mL by measuring optical density at 600 nm (OD_600_) and confirming counts through tenfold serial dilutions followed by duplicate plating on TSA and XLT-4 agar. This standardized inoculum was subsequently used for spiking ground chicken samples.

### 2.5. Preparation of Ground Chicken Samples and Enrichment Protocol

Ground chicken portions (32.5 g) were inoculated with the four-strain *Salmonella Typhimurium* cocktail to achieve target contamination levels ranging from −0.5 to 3.5 Log CFU/g. A negative control was included in each trial and confirmed using the BAX^®^ System Real-Time PCR assay. Each sample was placed in a sterile stomacher bag containing 162.5 mL of Buffered Peptone Water (BPW) and homogenized for 30 s. From the homogenate, 30 mL was transferred into a sterile filter bag, followed by the addition of 30 mL of pre-warmed (42 °C) MPN medium supplemented with novobiocin (40 mg/L). Samples were incubated at 42 °C for enrichment periods of 10 and 12 h.

### 2.6. SPR Assay

Enriched cultures were collected at 10 and 12 h for SPR analysis. For sample preparation, 900 μL of enriched culture was mixed with 100 μL of 10× PBST, heated at 85 °C for 10 min, and centrifuged at 1600 rpm for 10 min at room temperature. A 300 μL aliquot of the supernatant was transferred to an autosampler vial for SPR analysis. The SPR assay employed a direct format using immobilized MAb 1E10 to capture *Salmonella* flagellin. All SPR responses were corrected for non-specific refractive index effects. Specifically, the antibody-specific signal from the left channel was adjusted by subtracting the corresponding SPR signal from the right channel, which represented the non-specific refractive index. SPR responses, proportional to the amount of flagellin captured, were recorded as sensorgrams and analyzed using TraceDrawer software.

### 2.7. Most Probable Number (MPN) and Real-Time PCR Assay

Buffered Peptone Water (BPW) homogenates prepared as described in Section 2.5 were manually mixed for 30 s prior to analysis. Most Probable Number (MPN) sets were assembled in triplicate using five serial dilutions equivalent to 1, 0.1, 0.01, 0.001, and 0.0001 g of sample. For the initial dilution, 6 mL of homogenate was combined with 4 mL of BPW. Subsequent dilutions were prepared by transferring 1 mL from the preceding tube into 9 mL of BPW. All tubes were incubated at 37 °C for 24 h. The presence of *Salmonella* in each tube was confirmed using the BAX^®^ System Real-Time PCR assay. MPN values were calculated according to the MLG Appendix 2.05 reference table and expressed as Log MPN per gram of sample.

### 2.8. Experimental Design and Data Analysis

**Figure 1:**
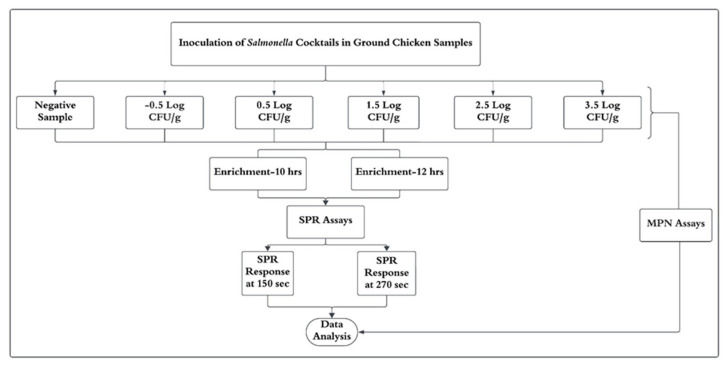
Experimental design illustrates six contamination levels, two enrichment periods, and two SPR response times. MPN analysis was performed for each sample. The entire experiment was repeated twelve times under consistent conditions.

A completely randomized design was employed, and the experiment was repeated twelve times under identical conditions (Figure 1). Each experiment included duplicate samples across six inoculation levels (−0.5 to 3.5 Log CFU/g), along with negative controls. Samples were subjected to two enrichment periods (10 and 12 h) and analyzed using SPR at two response times (150 and 270 s). Replicate SPR measurements were averaged, and the means were used for statistical analysis.

To account for potential sensor surface deterioration due to repeated regeneration, positive control was included in each run. SPR responses, measured in micro refractive index units (μRIU), were normalized by expressing each sample’s response as a percentage of the corresponding positive control. The normalized percentage positive response (%P) was calculated as:

%P = (Sample SPR Response/Positive Control SPR Response) × 100

Normalized %P values were utilized to enable comparison across experimental runs. For each enrichment duration, linear regression models were constructed by plotting contamination levels (Log CFU/g) against %P values. The coefficient of determination (R^2^) was calculated to assess the goodness of fit for each model. All statistical analyses were conducted using Microsoft Excel (Microsoft 365, 2021) and SAS software (version 9.4; SAS Institute Inc., Cary, NC, USA).

## 3. Results

### 3.1. Enumeration of Salmonella in Culture Cocktails

Composite cultures of *Salmonella Typhimurium* were prepared as described in Section 2.4 and enumerated on Tryptic Soy Agar (TSA) and Xylose-Lysine-Tergitol 4 (XLT-4) agar across twelve independent replicates. The mean count obtained on TSA was 8.90 Log CFU/mL with SD = 0.11 Log CFU/mL, while XLT-4 yielded a mean of 8.92 Log CFU/mL with SD = 0.11 Log CFU/mL. Dilutions for sample inoculation were calculated based on TSA enumeration, and ground chicken portions were spiked at varying concentrations as outlined in Section 2.8.

**Figure 2:**
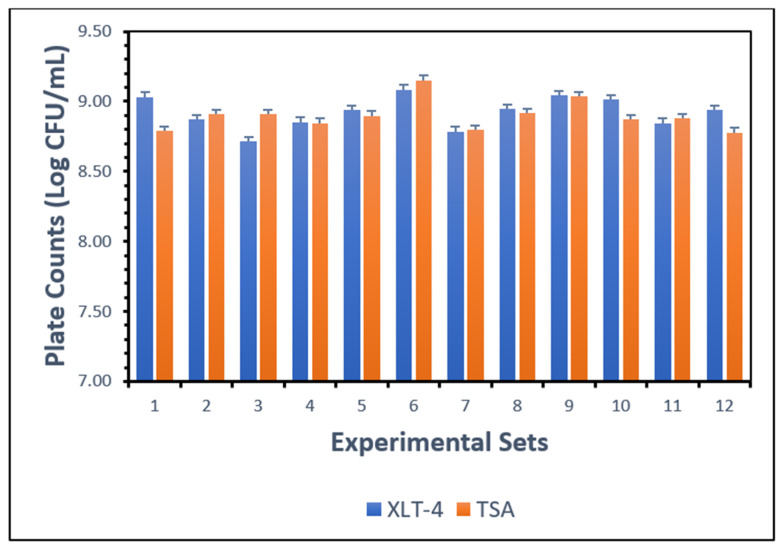
Comparison of TSA and XLT-4 agar for enumeration of *Salmonella Typhimurium* cultures.

Statistical analysis revealed no significant difference (*p* > 0.05) between TSA and XLT-4 within the same experimental set, indicating that both methods were equally effective for *Salmonella* enumeration under the conditions of this study (Figure 2). This comparison was performed using a paired *t*-test, which evaluates whether the mean difference between two related measurements (TSA vs. XLT-4 counts) is statistically significant. The lack of significance suggests strong agreement between the two media for enumeration.

In contrast, a significant difference (*p* < 0.05) was observed among different experimental sets conducted under similar conditions. This was assessed using one-way ANOVA, which determines whether there are statistically significant differences among the means of multiple groups. The observed variability is likely attributable to inconsistencies introduced during loop inoculation of colonies into TSB from stock plates, which can affect initial cell density and subsequent growth.

### 3.2. Optimization of SPR for Quantitative Determination of Salmonella

The SPR protocol was optimized to enhance accuracy and sensitivity while minimizing assay time. The optimized workflow consisted of two consecutive cycles, each comprising eight continuous assays: buffer control, positive control, and six ground chicken samples (including one negative control). Regeneration of the sensor surface was performed between assays to maintain performance.

**Figure 3:**
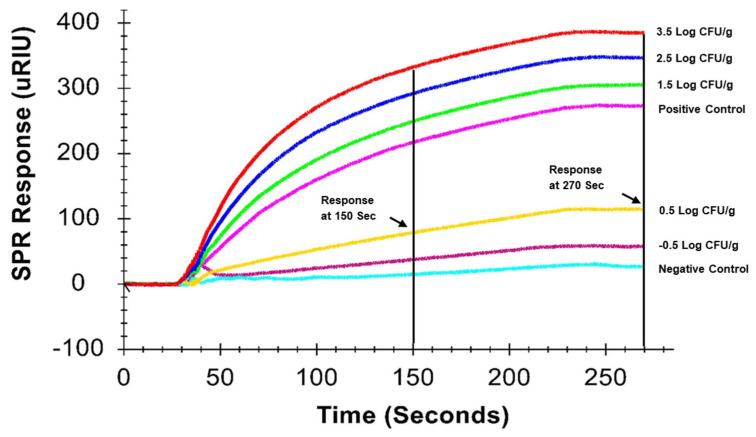
SPR sensorgrams showing responses at 150 and 270 s for ground chicken samples with different *Salmonella* contamination levels.

A representative sensorgram from one cycle of SPR analysis, illustrating responses for ground chicken samples inoculated at varying *Salmonella* contamination levels, is shown in Figure 3. Positive controls were included in each cycle to monitor sensor performance and to calculate normalized positive responses (%P) for all samples, as described in Section 2.8. SPR responses (μRIU) were extracted at 150 and 270 s and converted to %P for comparative analysis presented in Section 3.4.

### 3.3. Monitoring of SPR Sensor Surface Using Positive Controls

To ensure the reliability and accuracy of SPR measurements over multiple runs, the responses of positive controls obtained from the same sensor chip were monitored across 20 consecutive SPR runs. A gradual decline in SPR response was anticipated due to repeated regeneration of the sensor surface.

**Figure 4:**
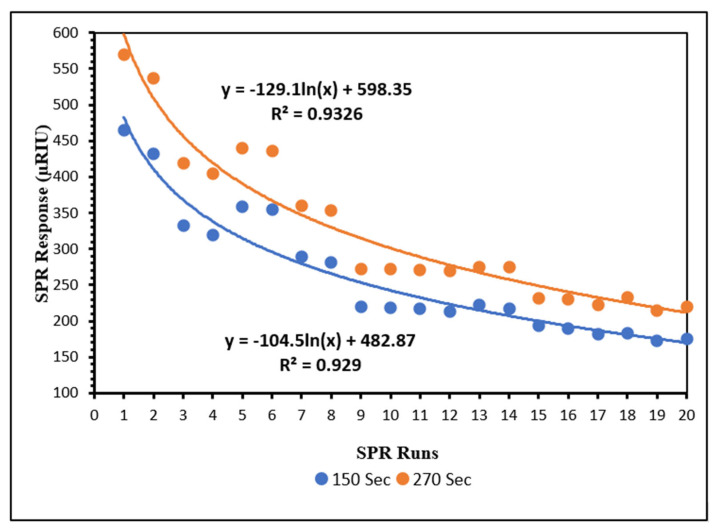
SPR responses of positive controls at 150 and 270 s over 20 consecutive runs.

As illustrated in Figure 4, the logarithmic model fits the data well, showing a strong negative relationship between SPR runs and μRIU (R^2^ ≥ 0.92). The negative coefficient for ln(x) indicates that μRIU decreases logarithmically as the number of SPR runs increases. The regeneration efficiency, calculated using the logarithmic regression model, demonstrates a progressive improvement across consecutive runs. After the first regeneration cycle (run 2), the efficiency is approximately 85%, indicating a substantial initial loss. However, efficiency increases rapidly in subsequent cycles, reaching over 93% by run 5 and exceeding 95% by run 8. Beyond this point, the improvement becomes incremental, with efficiency values gradually approaching 97% by run 20. This trend reflects a stabilization phase where the sensor retains nearly all of its previous performance after repeated regeneration, suggesting that the regeneration process becomes highly effective after the initial few cycles.

Regeneration, which involves disrupting antigen–antibody interactions using acidic or alkaline solutions, enables chip reuse, thereby reducing operational costs and extending sensor lifespan [44]. In this study, regeneration was performed using 10 mM glycine-HCl (pH 3.0) for 4 min, as previously described by Bhandari et al. [41], to remove antigen–antibody complexes and prepare the surface for subsequent assays.

Sensor surfaces were replaced when SPR responses fell below 200 μRIU, which was considered a low-response threshold. Most sensor chips supported more than 20 SPR runs, allowing analysis of at least 120 samples in duplicate per chip. To account for the gradual decline in sensor performance, normalized responses (%P) were calculated by expressing each sample’s response relative to the positive control response (Section 2.8), enabling quantitative comparison across different SPR runs.

### 3.4. Evaluation of SPR Quantitative Performance

Linear regression models were constructed to evaluate the quantitative performance of SPR for detecting *Salmonella Typhimurium* in ground chicken samples. The models plotted contamination levels (Log CFU/g) against normalized SPR responses (%P) for samples enriched for 10 and 12 h. SPR responses were recorded at 150 and 270 s and expressed as %P.

**Figure 5:**
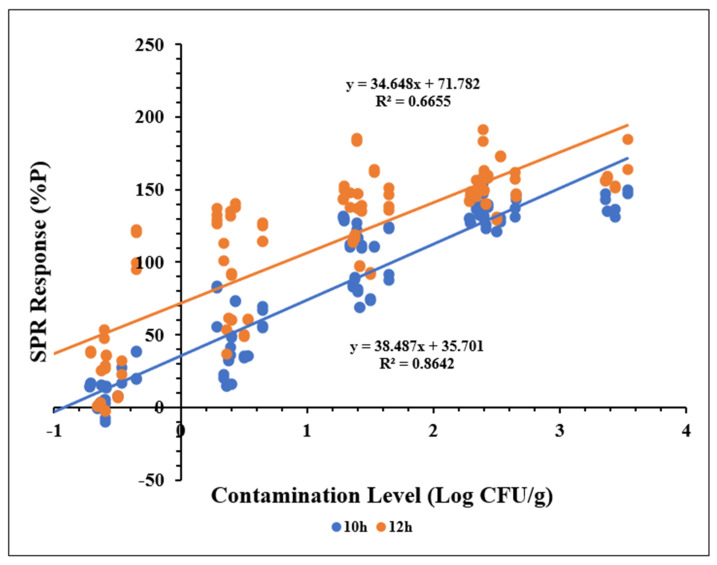
Linear correlation between SPR responses at 150 s and contamination levels (Log CFU/g) in ground chicken samples enriched for 10 and 12 h. Each data point represents the mean of two replicate measurements from the same sample.

**Figure 6:**
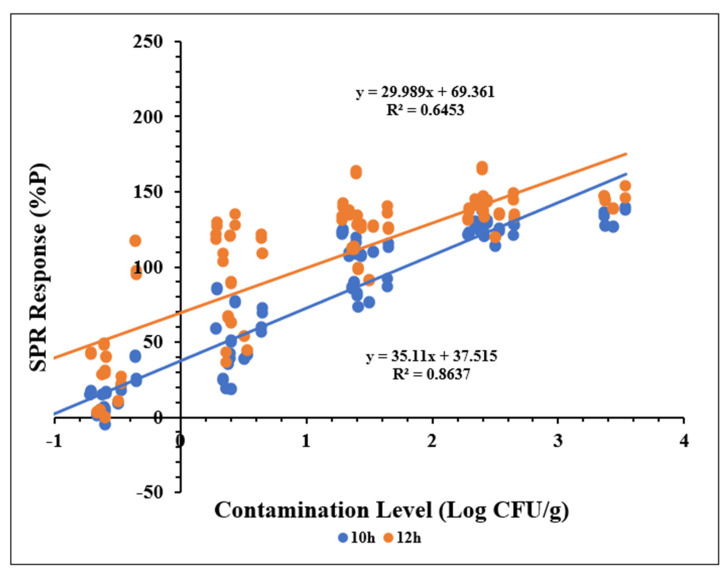
Linear correlation between SPR responses at 270 s and contamination levels (Log CFU/g) in ground chicken samples enriched for 10 and 12 h. Each data point represents the mean of two replicate measurements from the same sample.

As shown in Figure 5, SPR responses at 150 s exhibited a strong linear relationship with contamination levels for samples enriched for 10 h (R^2^ ≥ 0.86), indicating optimal performance within the range of −0.5 to 3.5 Log CFU/g. In contrast, the linear relationship was less pronounced for samples enriched for 12 h (R^2^ ≥ 0.64). Similar trends were observed for SPR responses at 270 s (Figure 6). These findings suggest that extending enrichment beyond 10 h does not improve quantitative accuracy. Preliminary trials) indicated that an 8 h enrichment period was insufficient to achieve detectable SPR responses.

No significant difference (*p* > 0.05) was observed between SPR responses at 150 and 270 s, indicating that extending the response time beyond 150 s does not enhance quantitative performance. Collectively, these results demonstrate that a 10 h enrichment period combined with a 150 s SPR response time provides optimal conditions for quantitative detection of *Salmonella Typhimurium* in ground chicken.

### 3.5. Comparison of SPR and MPN for Quantitative Determination of Salmonella

**Figure 7:**
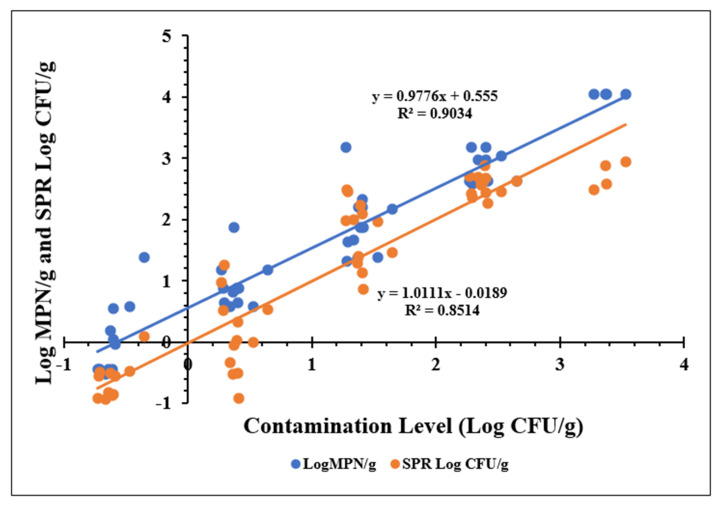
Comparison of SPR and MPN methods for quantifying *Salmonella Typhimurium* contamination levels in ground chicken samples.

Linear regression analysis was performed to evaluate the quantitative capability of the SPR-based method developed in this study relative to the conventional Most Probable Number (MPN) approach for determining *Salmonella Typhimurium* levels in ground chicken (Figure 7). The MPN procedure, described in Section 2.7, employed three replicates and five serial dilutions (15 tubes per sample), with positive tubes confirmed by real-time PCR following 24 h incubation. Log MPN/g values were derived using the MLG Appendix 2.05 reference table, whereas SPR-based Log CFU/g estimates were calculated using the regression equation obtained from 10 h enrichment data.

Results demonstrated a strong positive correlation between actual contamination levels (Log CFU/g) and predicted Log MPN/g values (R^2^ ≥ 0.90). Similarly, SPR-predicted Log CFU/g values correlated well with actual contamination levels (R^2^ ≥ 0.85), indicating consistency between the two quantification methods. Further examination of regression trends revealed that SPR provided slightly more accurate estimates, while MPN tended to overestimate contamination by approximately 0.5 Log CFU/g; however, this difference was not statistically significant.

Overall, these findings suggest that the SPR approach offers a reliable and efficient alternative to MPN for quantifying *Salmonella Typhimurium* in ground chicken, particularly when rapid and accurate results are required.

## 4. Discussion

*Salmonella* continues to be a major contributor to foodborne illnesses worldwide, despite growing attention to emerging pathogens in recent years [45]. In the United States, more than 20% of *Salmonella* infections are linked to poultry consumption [13]. Although several methods exist for quantitative detection of *Salmonella*, many are labor-intensive, time-consuming, and costly, underscoring the need for rapid, reliable, and cost-effective alternatives. Surface plasmon resonance (SPR) biosensors have gained considerable interest in foodborne pathogen detection due to their ability to monitor biomolecular interactions in real time, combined with simplicity and cost-effectiveness [30]. In this study, we developed an SPR-based method for quantitative detection of *Salmonella Typhimurium* in ground chicken and compared its performance with the industry-standard Most Probable Number (MPN) method.

The SPR assay utilized a well-characterized monoclonal antibody targeting *S. Typhimurium* flagellin, previously validated for its high specificity [41]. Earlier studies have shown that nanoparticle-assisted sandwich assays can significantly enhance SPR sensitivity, achieving signal intensities up to 14 times greater than those of direct assays [43]. Although the direct assay format was chosen in this study for its simplicity, future work integrating nanoparticle-enhanced sandwich assays could leverage their superior sensitivity to shorten enrichment times from 10 h to approximately 6–8 h.

Sensor surface regeneration is critical for enabling multiple SPR runs without replacing chips, thereby reducing costs. Various regeneration strategies have been reported, including EDTA/imidazole/SDS treatments for NTA-coated surfaces [46], acid and plasma treatments for silane-grafted gold chips [44], and piranha solution for APTES-functionalized chips [47]. In this study, regeneration was achieved using 10 mM glycine-HCl (pH 3.0) for 4 min, enabling over 20 reuse cycles per sensor chip. This approach allowed analysis of at least 120 samples in duplicate per chip. Monitoring positive controls during each run ensured normalization of responses across cycles, compensating for gradual signal decline due to repeated regeneration.

The sensitivity and accuracy of quantitative assays are commonly assessed using the limit of detection (LOD) and limit of quantification (LOQ). LOD indicates the lowest concentration of analyte that can be reliably detected, calculated as the mean control signal plus three times its standard deviation. Similarly, the LOQ is defined as the mean control signal plus ten times its standard deviation [48,49]. In this study, LOD and LOQ were determined from the SPR responses (%P) of negative samples (Mean = 2.76 ± 1.93, *n* = 24), resulting in calculated values of %P for LOD = 8.55 and LOQ = 22.06. Based on the linear regression models shown in Figure 5, these correspond to −0.71 Log CFU/g for LOD and −0.35 Log CFU/g for LOQ, both below 1 CFU/g. The combined use of BPW, MP media, and novobiocin likely contributed to this performance by promoting recovery of stressed cells, inhibiting competing microorganisms, and selectively targeting Gram-positive bacteria [50,51]. These findings align with previous reports demonstrating SPR detection of *S. Typhimurium* at concentrations below 1.0 Log CFU/g in leafy vegetables following BPW enrichment [41] and detection limits of 5.2 Log CFU/g in romaine lettuce without enrichment using magnetic nanoparticle concentration [43]. Detection limits in SPR assays are influenced by instrument sensitivity, antibody specificity, surface chemistry, and sample preparation methods [52].

Our results are consistent with prior studies on rapid quantification of *Salmonella* in poultry and other matrices, including immunomagnetic chemiluminescent assays for ground chicken [53], RT-PCR detection in pork and beef lymph nodes [54], and qPCR-based quantification in poultry and sheep tissues [17,23,55]. Collectively, these findings support the potential of SPR as a robust alternative to conventional methods for rapid and accurate quantification of *Salmonella Typhimurium* in food matrices.

Rapid quantitative techniques play a critical role in detecting pathogenic bacteria, thereby preventing widespread foodborne illness and offering significant potential for applications in food processing, preservation, and safety assessment [56]. Traditionally, the Most Probable Number (MPN) and standard plate count methods have been widely employed for bacterial quantification. However, the MPN method requires multiple serial dilutions and inoculation of numerous tubes, making it labor-intensive and time-consuming [17,22,23,57]. Additionally, prolonged incubation periods limit throughput in routine testing [22], and the method often overestimates bacterial counts [23]. Similarly, the standard plate count method involves isolation and biochemical confirmation of presumptive colonies, which is costly, laborious, and can take up to four days to yield definitive results [17,58].

Recent studies have explored alternative approaches to overcome these limitations. For instance, *Salmonella* Quant has been shown to provide faster and more reliable estimates compared to MPN, which frequently overpredicts bacterial loads and requires extended confirmation times [23]. Likewise, the BAX^®^ System SalQuant^®^ demonstrated comparable performance to MPN, suggesting its feasibility as an alternative for *Salmonella* quantification [54]. Multiple quantification methods—including MPN, BAX, GENE-UP, and PiLOT threshold test was compared, and it was reported that MPN and PiLOT-86 were the most accurate, followed by GENE-UP, PiLOT-50, and BAX [59]. Collectively, these findings indicate that although MPN remains the gold standard for bacterial quantification, its practical application is constrained by high costs and extended turnaround times, limiting its suitability for routine industrial or regulatory testing.

Based on the results of this study, SPR demonstrated strong potential as a reliable and efficient alternative to MPN for quantifying *Salmonella Typhimurium* in ground chicken. Table 1 summarizes key characteristics of four commonly used approaches: Real-Time PCR, Lateral Flow Immunoassay, MPN, and SPR. SPR biosensors enable real-time, label-free detection with high specificity, though they currently require specialized instrumentation and enrichment. Its ability to deliver rapid and accurate results positions SPR as a promising tool for food safety applications where timely and precise quantification is essential.

## 5. Conclusions

This study demonstrates the effectiveness and accuracy of surface plasmon resonance (SPR) for quantifying *Salmonella Typhimurium* in ground chicken. The optimal conditions identified were a 10 h enrichment period combined with an SPR measurement time of 150 s. Extending either the enrichment duration or the SPR measurement time did not improve sensitivity or accuracy. Comparatively, the Most Probable Number (MPN) method slightly overestimated contamination levels relative to SPR. Although MPN is widely regarded as the gold standard, its reliance on multiple serial dilutions and prolonged incubation makes it labor-intensive and impractical for routine industrial or regulatory testing.

The SPR method developed in this study provides results within approximately 14 h, including enrichment and assay time, whereas MPN requires a minimum of 24 h for incubation and PCR confirmation and may take up to a week for complete verification. Given its reduced labor requirements, faster turnaround, and cost-effectiveness, SPR represents a promising alternative to MPN for rapid and accurate quantification of *Salmonella Typhimurium* in food matrices.

## Figures and Tables

**Table 1:** Comparison of Rapid Detection Methods for *Salmonella*.

Feature/Method	Real-Time PCR	Lateral Flow Immunoassay	MPN	SPR Biosensor
Sensitivity	High	Moderate	High	High
Specificity	High	High	High	High
Quantitative	Yes (Ct values)	No	Yes (statistical estimate)	Yes (%P)
Enrichment Time	6–24 h	18–24 h	24–48 h	6–12 h
Time to Result	4–8 h post enrichment	20 min post enrichment	48–72 h total	1–2 h post enrichment
Limit of Detection	~1–10 CFU/25 g	~10^2^–10^3^ CFU/g	~1 CFU/g	Less than ~1 CFU/g
Cost	High	Low	Moderate	High
Field Deployable	Limited	Excellent	Poor	Limited

## Data Availability

The original contributions presented in this study are included in the article. Further inquiries can be directed to the corresponding author.
